# The Role of Quinine-Responsive Taste Receptor Family 2 in Airway Immune Defense and Chronic Rhinosinusitis

**DOI:** 10.3389/fimmu.2018.00624

**Published:** 2018-03-28

**Authors:** Alan D. Workman, Ivy W. Maina, Steven G. Brooks, Michael A. Kohanski, Beverly J. Cowart, Corrine Mansfield, David W. Kennedy, James N. Palmer, Nithin D. Adappa, Danielle R. Reed, Robert J. Lee, Noam A. Cohen

**Affiliations:** ^1^Department of Otorhinolaryngology: Head and Neck Surgery, University of Pennsylvania, Philadelphia, PA, United States; ^2^Monell Chemical Senses Center, Philadelphia, PA, United States; ^3^Department of Physiology, University of Pennsylvania, Philadelphia, PA, United States; ^4^Philadelphia Veterans Affairs Medical Center, Philadelphia, PA, United States

**Keywords:** quinine, taste receptor family 2, T1R, innate immunity, chronic rhinosinusitis, taste test

## Abstract

**Background:**

Bitter (T2R) and sweet (T1R) taste receptors in the airway are important in innate immune defense, and variations in taste receptor functionality in one T2R (T2R38) correlate with disease status and disease severity in chronic rhinosinusitis (CRS). Quinine is a bitter compound that is an agonist for several T2Rs also expressed on sinonasal cells, but not for T2R38. Because of this property, quinine may stimulate innate immune defense mechanisms in the airway, and functional differences in quinine perception may be reflective of disease status in CRS.

**Methods:**

Demographic and taste intensity data were collected prospectively from CRS patients and non-CRS control subjects. Sinonasal tissue from patients undergoing rhinologic surgery was also collected and grown at an air–liquid interface (ALI). Nitric oxide (NO) production and dynamic regulation of ciliary beat frequency in response to quinine stimulation were assessed *in vitro*.

**Results:**

Quinine reliably increased ciliary beat frequency and NO production in ALI cultures in a manner consistent with T2R activation (*p* < 0.01). Quinine taste intensity rating was performed in 328 CRS patients and 287 control subjects demonstrating that CRS with nasal polyps (CRSwNP) patients rated quinine as significantly less intense than did control subjects.

**Conclusion:**

Quinine stimulates airway innate immune defenses by increasing ciliary beat frequency and stimulating NO production in a manner fitting with T2R activation. Patient variability in quinine sensitivity is observed in taste intensity ratings, and gustatory quinine “insensitivity” is associated with CRSwNP status. Thus, taste tests for quinine may be a biomarker for CRSwNP, and topical quinine has therapeutic potential as a stimulant of innate defenses.

## Introduction

Chronic rhinosinusitis (CRS) is a complex syndrome with a multifactorial etiology of inflammation and infection (1). It affects up to 16% of the population, has a high economic burden (2), and is associated with significant morbidity. CRS is commonly divided into two subtypes based on the absence or presence of intranasal polyps. CRS without nasal polyps (CRSsNP) is most often characterized by a T helper type 1-mediated pattern of inflammation, while CRS with nasal polyps (CRSwNP) has greater propensity for a T helper type 2-mediated immune response (3). In recent years, studies have explored the role of airway taste receptors in sinonasal innate immunity, and how functional polymorphisms in these receptors may be implicated in the pathogenesis of CRS (4–8).

In humans, oral bitter and sweet taste perception is governed by G-protein-coupled receptors originally identified in taste bud type II cells (9, 10). Receptors belonging to taste receptor family 1 subtypes 2 and 3 (T1R2/T1R3) detect sweet compounds such as glucose and sucrose, while bitter taste receptors [taste receptor family 2 (T2Rs)] respond to a variety of bitter compounds including caffeine, denatonium, strychnine, and quinine (11). Stimulation of T2Rs activates the canonical taste signaling cascade involving phospholipase C β2 (PLCβ2) and transient receptor potential cation channel subfamily M member 5 (5). More recently, bitter and sweet receptors have been discovered in a variety of extraoral tissues including the brain, thyroid, pancreas, testes, and throughout the respiratory and GI tracts (12–14).

In the airway, taste receptors are present on a variety of cell types and have been shown to mediate several complementary components of innate immune defense. Ciliated sinonasal epithelial cells express T2R38 and respond to phenylthiocarbamide (PTC) and acyl-homoserine lactones, bitter compounds released by gram-negative bacteria such as *Pseudomonas aeruginosa*. Activation of T2R38 triggers an increase in intracellular calcium (Ca^2+^) yielding stimulation of nitric oxide synthase (NOS) with resultant production of intracellular nitric oxide (NO) (15). The NO, through cyclic GMP, increases ciliary beat frequency (CBF) and diffuses into the mucus layer where it has direct bactericidal activity (15–17). Similarly, solitary chemosensory cells (SCCs), rare epithelial cells that express both T1R2/3 and T2R receptors (18), also respond to bitter compounds secreted by bacteria in the upper airway. Stimulation of T2Rs on the surface of human SCCs by the bitter agonist denatonium elicits a calcium response that spreads *via* gap junctions to neighboring epithelial cells, triggering a release of pre-formed stores of antimicrobial peptides (4, 5). Currently, it is thought that the repertoire of T2Rs expressed on ciliated cells and the repertoire of T2Rs expressed on SCCs are mutually exclusive. Recent work demonstrates that the NO-producing T2R response is exclusively found in ciliated cells, while production of antimicrobial peptides is driven only by T2R’s on SCCs (5, 15, 19).

Stimulation of T1Rs by sweet compounds antagonizes the signal transduction of SCC T2Rs (5). It is hypothesized that during bacterial infection, airway mucus glucose levels are rapidly depleted due to bacterial consumption. This reduction in mucus glucose concentration is thought to remove the tonic activation of T1R2/3, thereby disinhibiting the signal transduction of T2Rs in response to bitter compounds secreted by pathogens. In addition, recent evidence demonstrates that in addition to glucose, bacterially produced d-amino acids (D-AAs) also activate the SCC sweet receptor attenuating the release of antimicrobial peptides (20). In this scenario, D-AAs are utilized by bacteria in biofilm dispersion (21, 22), but concomitantly inhibit host innate immunity.

Genetic variations in taste receptor functionality cause differential responsiveness in cells isolated from different individuals, and corresponding taste receptor function correlates with disease severity in CRS (5–7, 15, 23). Patients who are homozygous for the non-functional variant of T2R38 are more likely to require surgical intervention for CRS and are also more likely to develop gram-negative infection. Consequently, differences in oral taste perception and sensitivity can potentially indicate differences in the innate immune function of ciliated epithelial cells and SCCs. Recent work has shown that phenotypic taste tests with denatonium, a broad T2R agonist, and sucrose, a T1R2/3 agonist, can reflect clinical disease status in CRS and partially stratify control subjects and CRS patients (8). It is thought that patients with CRS possess hyporesponsive bitter taste receptors, rating denatonium as less bitter than controls, while also possessing hypersensitive sweet taste receptors, which compounds the reduced antimicrobial defense in response to sinonasal pathogens.

While recent studies have investigated the role of bitter taste receptors in CRS using the bitter agonist denatonium, other bitter agonists may help further elucidate the mechanisms by which some patients are predisposed to upper airway infection and inflammation. Differences in taste sensitivities may reveal corresponding functional alterations in airway immune defense. Quinine hydrochloride is a bitter agonist and alkaloid derivative isolated from the bark of the cinchona tree. It is known to stimulate nine T2Rs with varying efficacy (24), and quinine-sensitive T2Rs, including T2R4 (19) and T2R14 (25), are expressed in ciliated cells. In the present investigation, we demonstrate that quinine stimulates a NO antimicrobial response in the airway and that individuals in a CRS cohort are less sensitive to quinine in a phenotypic taste test. Functional differences in quinine perception may be predictive of functional responses in the airway and CRS pathology.

## Materials and Methods

### Reagents

4-Amino-5-methylamino-2′,7′-difluorescein (DAF-FM) was purchased from Invitrogen (Carlsbad CA, USA), and cPTIO was from Cayman Chemical (Ann Arbor, MI, USA). All other reagents, including quinine HCl, were obtained from Sigma-Aldrich unless otherwise indicated. Stock solutions of DAF-FM and cPTIO were made at 1:1,000 dilution in DMSO and were made fresh daily. All air–liquid interface (ALI) experiments were performed with Dulbecco’s PBS (1.8 mM Ca^2+^) on the apical surface of the cultures, while the basolateral side was bathed in a modified HEPES-buffered Hank’s Balanced Salt Solution (HBSS) with 1× MEM amino acids to provide a source of arginine (0.6 mM) for the production of NO.

### Patient Recruitment

With University of Pennsylvania Institutional Review Board approval and patient consent, adult patients meeting objective and subjective guidelines for the diagnosis of CRS based on the clinical practice guidelines for sinusitis from the Academy of Otolaryngology—Head and Neck Surgery (26) were recruited for the study. Only immune competent CRS patients older than 18 years with endoscopic evidence of sinonasal inflammation were included, and all patients had to undergo sinonasal surgery for their disease. Exclusion criteria included individuals with genetic disorders of mucociliary clearance, history of chemotherapy, immune deficiencies, or granulomatous disease. Demographic data, including age, gender, and race, were collected based on self-report, and information regarding medical history and use of nasal therapeutics was also collected. This information included a history of previous surgeries for chronic sinusitis, antibiotic use in the month prior to presentation, steroid and nasal irrigation use, diabetes and smoking status, and sinonasal outcomes test (SNOT-22) scores. Control subjects were family members of twins recruited in Twinsburg, Ohio, at the 2015 Twins Days Festival. Control subjects with prior sinus surgery or a sinus infection treated with antibiotics or steroids in the previous 6 months were excluded from the analysis.

### ALI Culture

Sinonasal specimens for culture were obtained during sinonasal surgery and transported to the laboratory on ice for growth into an ALI culture. The growth of human nasal epithelial cells at an ALI has been previously well described (15). Briefly, human sinonasal epithelial cells were enzymatically dissociated and grown with medium containing DMEM/Hem’s F-12 and bronchial epithelial-based medium (Lonza, Walkerville, MD, USA), in addition to 100 U/ml penicillin and 100 µg/ml streptomycin for 7 days. Following this, cells were trypsinized and placed on porous polyester membranes in transwell cell culture inserts (Transwell-clear, 12-mm diameter, 0.4-µm pores; Corning). These inserts were coated with 100 µl of coating solution (BSA 0.1 mg/ml; Sigma-Aldrich), type 1 bovine collagen (30 µg/ml; BD), and fibronectin (10 µg/ml; BD) in LHC basal medium (Invitrogen). After 5 days, the apical compartment was cleared, and the epithelium was allowed to differentiate using a medium of 1:1 DMEM (Invitrogen) and BEBM (Lonza), with the Clonetics complements for hEGF (0.5 ng/ml), epinephrine (5 µg/ml), hydrocortisone (0.5 µg/ml), BPE (0.13 mg/ml), insulin (5 µg/ml), triiodothyronine (6.5 µg/ml), and transferrin (0.5 µg/ml), supplemented with 100 UI/ml penicillin, 100 µg/ml streptomycin, 0.1 nM retinoic acid (Sigma-Aldrich), and 10% FBS (Sigma-Aldrich) in the basal compartment.

Prior to experiments, transwell inserts were removed from the basolateral media and placed in a new transwell with 600 µl of HBSS with vitamins. The apical side of the transwell was washed once with 250 µl of PBS, and then 30 µl of PBS was added to the surface.

### Live-Cell Imaging With DAF-FM in ALI Cultures

4-Amino-5-methylamino-2′,7′-difluorescein imaging was performed using a 488-nm argon laser line of a Fluoview FV1000 laser scanning confocal system and IX-81 microscope (Olympus). Cells were loaded with DAF-FM as previously described in ALI experiments (15). Briefly, cells were loaded with DAF-FM in PBS containing 10 µM DAF-FM diacetate in addition to 5 μM carboxy PTIO, a cell-permeant NO scavenger (apical side). After 30 min of incubation, apical surfaces of cultures were washed with PBS to remove all unloaded DAF-FM and cPTIO. Cells were incubated for 15 min to optimize dye retention, and then DAF-FM fluorescent images were acquired at 5-s intervals (10 μs/pixel, 512 × 512 resolution). Microscope and software settings were identical for each experiment.

### CBF Imaging and Analysis

Utilizing the 20× objective on an inverted microscope (Leica Microsystems, Bannockburn, IL, USA), CBF measurements were obtained from individual cultures. A model A602f-2 Basler area scan high-speed monochromatic digital video camera (Basler AG, Ahrensburg, Germany) captured images at 100 frames/s with a resolution of 650 × 480 pixels. Images from the camera were individually sampled by an acquisition board (National Instruments, Austin, TX, USA) on a Dell XPS 710 workstation (Dell, Inc., Round Rock, TX, USA) running the Windows XP Professional operating system (Microsoft, Redmond, WA, USA). Sisson-Ammons Video Analysis software (National Instruments), that is specialized to quantify CBF (27) by performing a whole-field analysis of the ciliated apical surface of the cultures, was used to analyze images, reporting a CBF that is the arithmetic mean of all of the cilia in the video field. A baseline CBF was calculated as an average of the first four CBF measurements in each culture prior to quinine application. 30 µl of quinine solution to be tested was introduced to the apical surface of the ALI culture at *t* = 0, giving a total apical fluid volume of 60 µl (30 µl quinine and 30 µl PBS). CBF was measured every 15 s after compound addition for 10 min.

### Taste Test

Subjects tasted and rated two, 5-ml samples of several taste solutions, including 0.35 M sucrose, 0.25 M sodium chloride (NaCl), and 56 µM quinine HCl. Concentrations were of moderate intensity and detectable by most individuals based on previous research (28). Water used to dilute the solutions was obtained from a Millipore purification system (Billerica, MA, USA). All taste compounds were obtained from Sigma Life Science (St. Louis, MO, USA). Solutions were prepared at the Monell Chemical Senses Center in Philadelphia, PA, USA, dispensed into glass vials, tightly capped, packaged, and taken to the testing location. All samples were presented twice in a fixed order. Subjects also were provided with a 25.3 fluid oz bottle of spring water from Whole Foods Market and a plastic cup for expectoration. Each subject rinsed their mouth once with water before and after tasting each solution. Subjects were asked to rate the intensity of each taste stimulus on a 13-point, validated category scale with equidistant verbal descriptors. These descriptors varied from “Extremely intense” (#12), “Very intense” (#9), “Moderately intense” (#6), “Slightly intense” (#3), and “No intensity at all” (#0). This scale has been previously used in a number of clinical studies at the Monell Chemical Senses Center (8, 29). Taste intensity ratings for the two trials of each tastant were averaged. In addition, a quinine/sucrose combination score was obtained by dividing each patient’s quinine and sucrose scores by their overall taste test score for all modalities, eliminating subjective differences in scaling behavior. Then, these proportions were combined by taking one minus the sucrose proportion and adding that value to the quinine proportion.

### Statistics

All statistical analyses were performed using GraphPad Prism or Stata/SE 14.2. All tests were two tailed, and *p* < 0.050 was the cutoff for statistical significance. For taste intensity testing, Fisher’s exact test was utilized to compare CRS vs. control groups for dichotomous and categorical covariates. Comparisons of groups on continuous variables, including age and SNOT-22 scores, were conducted using general linear models. To obtain an unbiased estimate of the difference in taste intensity ratings between CRS and control subjects, propensity score methods were used. In propensity score modeling, a propensity score for each participant is calculated based on their probability of being in the “treatment” group conditional on covariates using a logistic regression. In this study, the “treatment” group was arbitrarily considered to be the control group. Control and CRS patients’ taste test scores are then compared with their propensity scores added as weights to the linear regression model. This method allows for an unbiased estimate of the average difference in taste intensity ratings to be calculated.

## Results

### Quinine Stimulation Increases NO Production in Primary Human Sinonasal ALI Cultures *via* a Pathway Supporting T2R Activation

**Figure 1:**
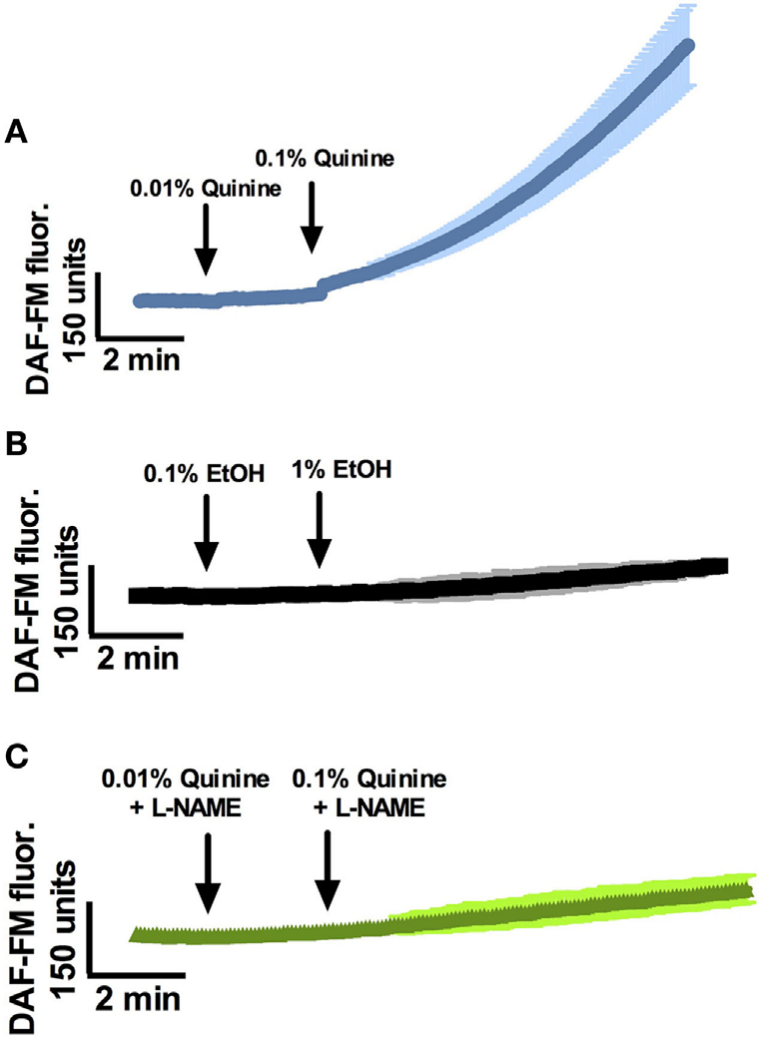
Quinine stimulates nitric oxide (NO) responses in a dose-dependent manner in sinonasal air–liquid interfaces. **(A)** NO responses to 0.01% and 0.1% quinine, **(B)** EtOH vehicle, and **(C)** quinine in the presence of l-N^G^-nitroarginine methyl ester (L-NAME) (*n* = 3–6 cultures). NO response to quinine is significantly greater than response to EtOH vehicle or quinine plus L-NAME after a period of 10 min, *p* < 0.01.

At least two quinine-responsive T2Rs are expressed in human sinonasal epithelial cells and in ALI culture (19, 25), and cultures used for the following experiments were shown to express both T2R4 and T2R14 by polymerase chain reaction (Figure S1 in Supplementary Material). Prior work with T2R38 has revealed a T2R stimulation pathway in ciliated cells that results in NO production that has antimicrobial activity (15). To test the hypothesis that stimulation with quinine HCl results in NO production in ciliated cells, we measured cellular NO production in response to quinine stimulation using the fluorescent probe DAF-FM. DAF-FM reacts with NO-derived nitrogen species to form a fluorescent benzotriazole (15). By using confocal imaging, NO production increases were calculated based on changes in fluorescence intensity. It is important to note that changes in intensity are noted in “DAF-FM units,” which can only be utilized in comparison to a control condition or an alternate condition, and as absolute DAF-FM unit values may differ between experiments. Overall, stimulation of ALI’s with quinine HCl + 0.1% EtOH causes dose-dependent (0.01 and 0.1%) increases in NO production over the course of 15 min (Figure 1, *n* = 6 cultures). Average DAF-FM fluorescence increase was 913.4 ± 125.9 units for quinine-stimulated cultures, compared to a 125.9 ± 6.4 unit increase for cultures stimulated with 0.1% EtOH vehicle (*n* = 3 cultures, *p* < 0.01). To demonstrate that increases in DAF-FM were due to the presence of NO and not a different reactive O_2_ or nitrogen species, ALI cultures were stimulated in the presence of the NOS inhibitor l-N^G^-nitroarginine methyl ester (L-NAME). NO DAF-FM fluorescence in response to quinine was blocked by L-NAME (*n* = 3 cultures).

**Figure 2:**
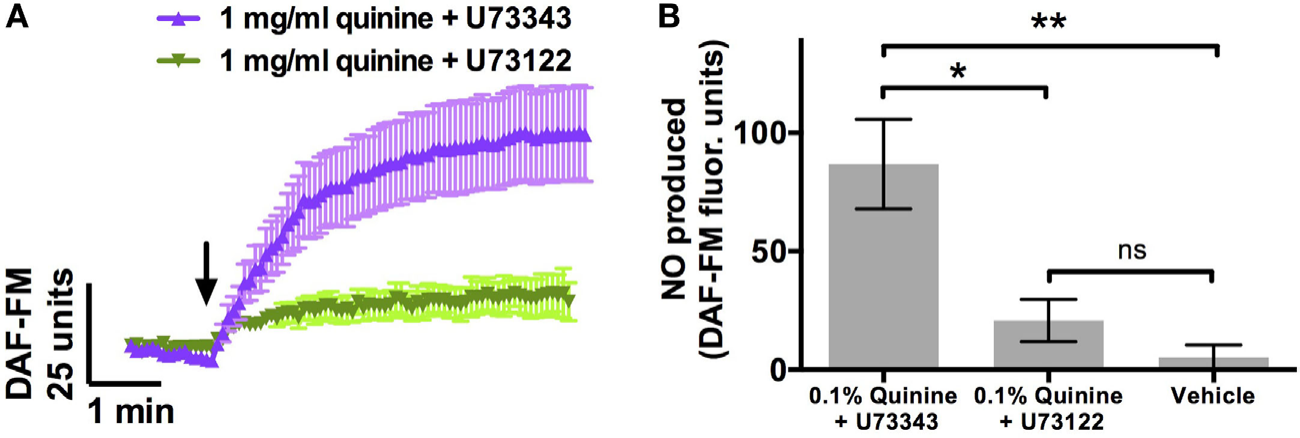
Quinine-induced nitric oxide (NO) release [as assessed by 4-amino-5-methylamino-2′,7′-difluorescein (DAF-FM)] is significantly attenuated by U73122, a phospholipase C β2 blocker, which is necessary for T2R-mediated signaling. This attenuation is not observed with incubation with U73343, an inactive analog of U73122 (*n* = 6–9 cultures). **(A)** NO release over a period of 5 min, and **(B)** maximal NO release during that same period. *p < 0.05, **p < 0.01.

Phospholipase C β2 is a necessary component of the Ca^2+^ cascade involved in T2R signaling (15). PLCβ2 knockout mice cannot distinguish between quinine and water (9). To confirm that the NO increase observed in response to quinine involved this pathway, cultures were incubated with the PLCβ2 blocker U73122 (*n* = 9) or the inactive analogue U73343 (*n* = 6) prior to DAF-FM experiments. Cultures incubated with the active PLCβ2 blocker demonstrated significant attenuation of the increase in DAF-FM fluorescence comparable to control experiments, while those incubated with the inactive analog demonstrated robust NO production (*p* < 0.01, one-way ANOVA; Figure 2). Thus, quinine-dependent NO production was inhibited by blocking PLCβ2 activation of downstream Ca^2+^ signaling.

### Quinine Increases CBF in Sinonasal ALIs

**Figure 3:**
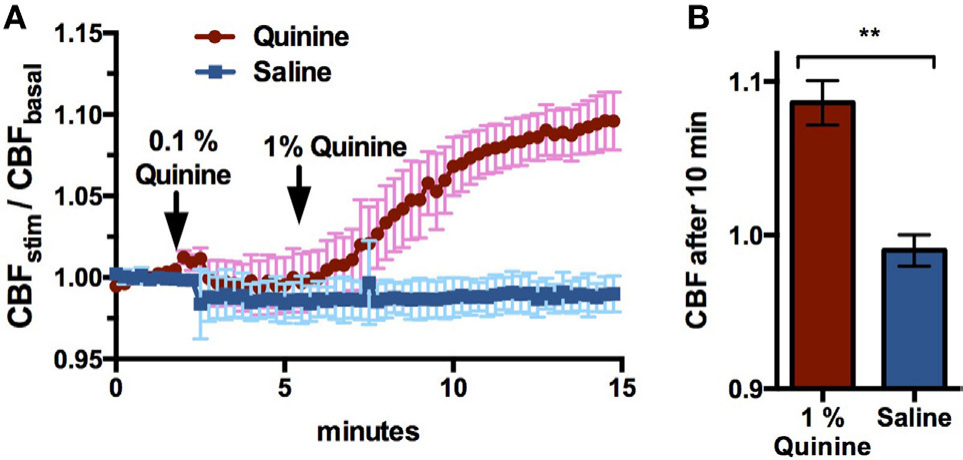
1% Quinine hydrochloride application significantly increases ciliary beat frequency (CBF) in sinonasal air–liquid interface cultures, compared to control saline (*n* = 6–7 cultures). Increases are reported in stimulated CBF/baseline CBF. **(A)** CBF increases over a 15 min period, and **(B)** a snapshot of CBF change after 10 min. **p < 0.01.

Cellular NO production in response to T2R stimulation causes NO-dependent increases in CBF and resulting mucociliary clearance (15). To show that quinine stimulates CBF, ciliary beating in ALI cultures was recorded before, during, and after the application of 0.1 and 1% quinine hydrochloride, and CBF increases were compared to those observed in control cultures following application of saline. Application of 1% quinine hydrochloride resulted in an increase in CBF over 10 min, with CBF in quinine-stimulated cultures increasing 9.6 ± 4.7%, while CBF in control cultures decreased by 1.0 ± 1.1% (*n* = 6–7 cultures, *p* < 0.01, Figure 3).

### NO Production in Response to Quinine Is T2R38 Independent

**Figure 4:**
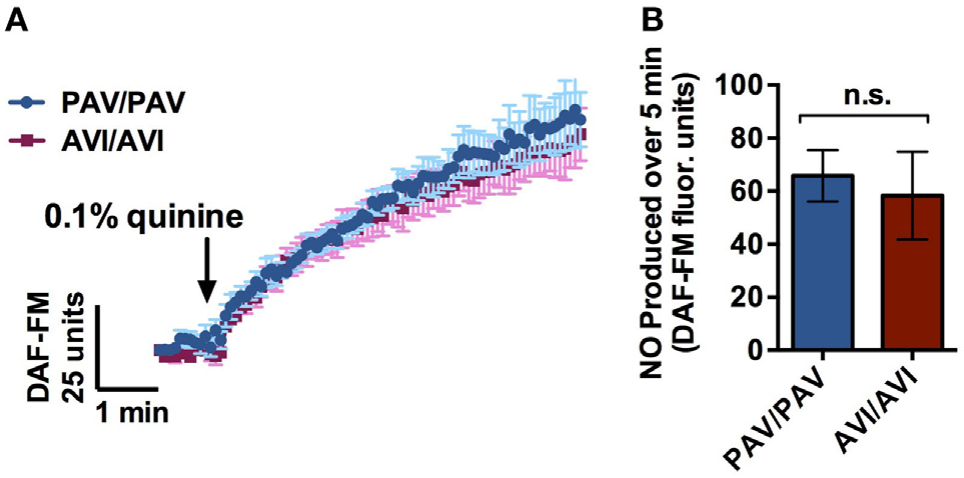
Air–liquid interface (ALI)’s with a genotype homozygous for the functional form of the T2R38 receptor (PAV/PAV) or a genotype homozygous for the non-functional form of the receptor (AVI/AVI) demonstrated no significant differences in 4-amino-5-methylamino-2′,7′-difluorescein (DAF-FM) fluorescence intensity in response to 0.1% quinine over a period of 7 min (*n* = 6 cultures per condition). **(A)** Nitric oxide (NO) release over a period of 5 min, and **(B)** maximal NO release during that same period.

T2R38 functionality varies by patient genotype, with patients having zero, one, or two copies of the functional form of the receptor (PAV), with the non-functional form referred to as AVI. This results in a continuum of PTC responsiveness *in vitro* and in the corresponding production of NO as assessed by DAF-FM (15). Quinine does not stimulate T2R38, but instead stimulates other distinct T2Rs (30). To confirm, as a control, that *TAS2R38* genotype (gene encoding T2R38 receptor) did not have an effect on quinine-induced NO responses, cultures from PAV/PAV patients (homozygous for the functional T2R38 receptor) and AVI/AVI patients (homozygous for the non-functional T2R38 receptor) were obtained and tested for DAF-FM fluorescence in the presence of 0.1% quinine (*n* = 6 cultures per condition; Figure 4). No differences were observed in NO production over a period of 7 min regardless of TAS2R38 genotype status.

In a separate analysis of ALI cultures from three individual patients, NO production as a result of stimulation with 0.01 and 0.1% quinine varies by individual. Each individual patient was genotyped at 4 SNPs in TAS2R4 and TAS2R14 gene transcripts (Figure S2 in Supplementary Material). While there is a variation observed in individual genotypes, there is not yet enough known about the biology of these receptors and how these polymorphisms translate to receptor function.

### Quinine Taste Testing in Patients With CRS

328 CRS patients and 287 control subjects were recruited to the study, and demographic and clinical data are reported in Table 1. Propensity score modeling was used to balance the groups, as CRS and control patients differed on several variables, including sex, age, and smoking status. These variables were included in the propensity score model to eliminate the possibility of confounders on our outcome, the taste test results. Once the propensity scores were determined and the groups were balanced, we calculated an unbiased estimate of difference in taste intensity ratings between each group, including control patients, CRSsNP, and CRSwNP. This propensity score matching created unbiased score estimates, eliminating the possible confounding of sex, age, and smoking status. These taste intensity ratings are shown in Table 2 as both raw intensity ratings and estimated mean differences from the propensity score model. While no significant differences in the perceived intensity of quinine were observed between controls and CRSsNP patients, patients with CRSwNP perceived quinine to be significantly less intense than did controls (mean difference, −0.89 ± 0.21, *p* < 0.001). Because T1R (sweet) taste receptors have an opposing effect to T2Rs, attenuating innate immune responses (5, 20), taste intensity perception of sucrose was also evaluated. These sucrose results have been previously reported by our group (8), with both CRSsNP and CRSwNP patients perceiving sucrose to be significantly more intense than do controls. NaCl sensitivity (non-bitter, non-sweet) did not differ between control and CRS subjects.

**Table 1:** Demographic data for chronic rhinosinusitis (CRS) and control subjects.

	CRS	Control	*p*
Total enrollment	328	287	
Male, *N* (%)	199 (61)	121 (42)	<0.001
Race, *N* (%)			0.787
White	289 (88.1)	249 (87.1)	
Non-white	39 (11.9)	38 (12.9)	
Ethnicity, *N* (%)			0.003
Hispanic	3 (1)	15 (5.2)	–
Non-Hispanic	325 (99.1)	272 (94.8)	–
Smoker, *N* (%)			<0.001
Never	197 (60.1)	236 (82.2)	
Ever	131 (39.9)	51 (17.8)	
Asthma, *N* (%)	162 (49.4)	41 (14.4)	<0.001
Nasal Polyps, *N* (%)	186 (56.7)	–	–
Prior FESS, *N* (%)			
Primary	131 (39.9)	–	–
Revision	197 (60.1)	–	–
	Mean (SD)	Mean (SD)	
Age at enrollment	48.6 (15.3)	40.4 (15.4)	<0.001
Sinonasal outcomes test-22	46.6 (22.2)	10.6 (12.2)	<0.001
BMI	28.0 (5.3)	–	
Lund-Mackay CT	12.3 (6.0)	–	

**Table 2:** Left: Taste intensity testing results (raw mean score) in control, CRSsNP, and CRSwNP patients. Right: Propensity score modeling-adjusted differences between control and CRS groups reported as adjusted difference (standard error).

	Raw taste intensity scores			Propensity score adjusted differences			
	Control	CRSsNP	CRSwNP	Control vs. CRSsNP	*p*	Control vs. CRSwNP	*p*
Quinine	3.26	2.69	2.22	0.29 (0.24)	0.221	**0.89 (0.21)**	**<0.001**
Sucrose	5.99	6.43	6.44	−**0.65 (0.25)**	**0.011**	−**0.65 (0.23)**	**0.005**
NaCl	6.67	6.44	6.68	−0.06 (0.27)	0.824	−0.22 (0.23)	0.356
Comb. score	−6.67	−10.02	−13.77	**4.18 (1.10)**	**<0.001**	**5.94 (1.01)**	**<0.001**

*Significant values are shown in bold*.

As T1R and T2R receptors have opposing downstream effects in airway innate immune defense (5), we also examined a quinine/sucrose combination score that reflects the additive effect of these differing sensitivities. Overall, large differences were observed between CRS and control subjects (mean difference: CRSsNP, 4.18 ± 1.10, *p* < 0.001 and CRSwNP, 5.94 ± 1.01, *p* < 0.001). Thus, the inclusion of both bitter (quinine) and sweet (sucrose) taste sensitivities results in augmented observed differences between the control and CRS cohorts.

## Discussion

Nitric oxide plays a protective role in airway innate immune defense. When produced in response to bitter taste receptor stimulation of T2Rs on ciliated cells, it speeds up ciliary beating and also diffuses into the airway where it directly damages bacterial membranes, enzymes, and DNA (15). It is thought that this bactericidal activity is a means of preventing commonly encountered pathogens from excessively proliferating in the sinonasal tract (15). In addition, there is likely a significant microbial contribution to CRS and the disease tends to run in families, suggesting a genetic component to the disease (31). Genetic variation in bitter and sweet taste receptors has been correlated with disease status and disease severity in CRS (4–6, 8), and here, we show that quinine is an additional compound that stimulates airway T2Rs and elicits taste differences in CRS and control subjects.

In response to 0.1% quinine HCl, there was a rapid increase in intracellular NO in all patient ALI cultures evaluated, increasing over the course of 10 min. Similar increases were not observed in control ALI’s receiving treatment with vehicle. CBF can be increased in part through cellular NO production as well, and stimulatory CBF changes were observed following quinine addition in ALI culture. As quinine is a promiscuous compound that activates several T2Rs, no single genetic polymorphism would entirely attenuate NO production in response to quinine, but several receptor polymorphisms may have compounding effects. Interestingly, quinine stimulates at least nine T2Rs in the same concentration range, in contrast to denatonium benzoate, which has a concentration range of T2R activation spanning 5 orders of magnitude (11).

Several investigations have examined the effect of quinine on the lower airway, particularly in inflammatory modulation and bronchoconstriction. In mouse asthma models, pretreatment of animals with quinine reduces infiltration of inflammatory cells and also attenuates excessive mucus accumulation (32). Specifically, a dose-dependent reduction of neutrophil and other immune cell recruitment was observed in a chemotactic gradient with quinine administration. Other studies have demonstrated a reduction in bronchoconstriction and airway remodeling with quinine, particularly due to smooth muscle relaxation (33). These effects are at least partially T2R and NO dependent, as they are inhibited by L-NAME and U73122 (19, 25, 34). The immunomodulatory effects of quinine-responsive T2Rs throughout the airway are still being fully elucidated, but utilizing quinine as an agonist to stimulate innate immune defenses may have therapeutic potential in CRS and other respiratory diseases.

Common genetic variants in a cluster of bitter taste receptor genes on chromosome 12 appear to have a robust contribution to perception of quinine taste intensity (35). Quinine taste sensitivity also appears to have been selected independently in some world populations, particularly for low concentration levels of quinine (36). Concentrations of bitter microbial products in the airway are at correspondingly low concentrations (15), and these differences in quinine taste perception at these concentrations may be reflective of varying responses of these bitter taste receptors on the tongue and in the airway. In our investigation, we demonstrate that patients with CRSwNP are significantly less sensitive to the bitter taste of quinine. This complements previous data showing that the patients with the other CRS phenotype, CRSsNP, are significantly less sensitive to denatonium, a broad T2R agonist (8). However, denatonium is detected by T2Rs located on SCC’s, and the SCC downstream response of T2R stimulation is an increase in antimicrobial peptide secretion, while NO release is more characteristic of a ciliated cell T2R response. These two bitter products, denatonium and quinine, thus elicit different physiologic responses and may demonstrate unique T2R contributions to the two broad types of CRS (with and without polyps). Previous work also shows that patients with CRS irrespective of phenotype are more sensitive to sucrose, which is a T1R (sweet taste receptor) agonist. As T1R stimulation opposes the action of T2R stimulation in SCCs, these patients are thought to inhibit T2R function at lower airway glucose concentrations, such as during an early airway infection, due to this high affinity. Taste testing that aggregates differences in multiple bitter and sweet taste products that stimulate different receptors may prove useful in achieving improved patient stratification. Further work is necessary to optimize compound concentrations that most accurately reflect taste receptor affinities in the sinonasal tract.

Quinine is a broad T2R agonist that stimulates NO responses, with resultant bactericidal activity and increases in mucociliary clearance. The ability of quinine and other bitter tastants to harness innate immune defense mechanisms may have therapeutic potential as topical therapies for sinonasal diseases. Beyond this, inexpensive phenotypic taste tests for quinine and other bitter and sweet compounds can potentially predict airway taste receptor variation and associated predisposition to infectious or inflammatory disorders. A predictive taste test with improved performance parameters may have utility in identifying patients at risk of CRS or refractory disease and also may prove useful in identifying candidates for aggressive surgical or medical management of their disease.

## Ethics Statement

This study was carried out in accordance with the recommendations of the Institutional Review Board at the University of Pennsylvania with written informed consent from all subjects. All subjects gave written informed consent in accordance with the Declaration of Helsinki. The protocol was approved by the University of Pennsylvania Institutional Review Board.

## Author Contributions

AW, BC, CM, DK, JP, NA, DR, RL, and NC contributed substantially to the conception and design of the work over a period of several years; AW, IM, SB, MK, BC, CM, DR, and RL acquired and interpreted data for the work; AW, IM, SB, RL, and NC drafted the work and approved the final version for publication while agreeing to be accountable for all aspects of the work; MK, BC, CM, DK, JP, NA, and DR revised the work critically and approved the final version for publication while agreeing to be accountable for all aspects of the work.

## Conflict of Interest Statement

The senior author (NC) and penultimate authors (RL and DR) have a patent pending “Therapy and Diagnostics for Respiratory Infection.” The other authors declare no competing interests.
