# Supplementary material for: The Role of Quinine-Responsive Taste Receptor Family 2 in Airway Immune Defense and Chronic Rhinosinusitis
Source: Front Immunol. 2018 Mar 28;9:624. doi: 10.3389/fimmu.2018.00624 (PMC5882797; doi:10.3389/fimmu.2018.00624)
Supplement: Supplementary file 1 [file data_sheet_1.docx]

*Supplemental Figure 1: Polymerase chain reaction (PCR) of select TAS2R gene transcripts in sinonasal ALI cultures. Negative controls are shown adjacent, denoted with “-“. TAS2R1 (790bp); TAS2R3 (697bp); TAS2R4 (726bp); TAS2R5 (668bp), TAS2R13 (743bp), TAS2R14 (771bp), TAS2R16 (449bp), TAS2R38 (766bp).*

| **TAS2R** | **SNP** | **Patient 2766** | **Patient 1231** | **Patient 2772** |
| --- | --- | --- | --- | --- |
| 4 | rs2233998 | C:T |  | T:T |
| 4 | rs2234001 | C:G |  | G:G |
| 4 | rs2234002 | A:G | A:G | G:G |
| 14 | rs111614880 | T:T | T:T | T:T |

*Supplemental Figure 2: (Left) Quinine stimulated NO responses in 3 individual patients (n=3-6 individual ALI cultures per patient). (Right) SNP sequencing at several loci in TAS2R4 and TAS2R14. T2R4 and T2R14 are both quinine-responsive T2Rs in ciliated cells. Functional consequences of genetic polymorphisms are currently unknown.*
